# 6-Minute Walk Test: Exploring Factors Influencing Perceived Intensity in Older Patients Undergoing Cardiac Rehabilitation—A Qualitative Study

**DOI:** 10.3390/healthcare13070735

**Published:** 2025-03-26

**Authors:** Gilbert Büsching, Jean-Paul Schmid

**Affiliations:** 1 Medical Therapeutic Departement, Klinik Barmelweid AG, 5017 Barmelweid, Switzerland; 2 Barmelweid Academy, Klinik Barmelweid AG, 5017 Barmelweid, Switzerland; jean-paul.schmid@kliniken-valens.ch; 3 Department Internal Medicine, Klinik Gais AG, 9056 Gais, Switzerland; 4 Cardiovascular Prevention and Rehabilitation Unit, University Hospital (Inselspital), University of Bern, 3010 Bern, Switzerland

**Keywords:** cardiovascular rehabilitation, coronary heart disease, six-minute walk test, rating of perceived exertion, Borg Scale

## Abstract

**Background/Objectives**: In cardiac rehabilitation, the 6-minute walk test (6-MWT) is a standard assessment of initial evaluation. It measures walking distance as a surrogate of submaximal physical performance. Thereby, a subjective rating of perceived exertion, assessed by the Borg Scale, plays an important role. It has been observed that patients with coronary heart disease often rate themselves lower than the person supervising the test. Since this discrepancy might lead to inadequate exercise prescription, this study aims to explore reasons for low self-rating. **Methods:** In a qualitative study, influencing factors for low self-rating were collected from patients using interviews and questionnaires and from a focus group of physiotherapists. The evaluation was based on the grounded theory. **Results:** Self-image after retirement emerged as the central factor, as it shaped patients’ behavior during the 6-MWT and their subsequent self-assessment. Additionally, five different categories of causal conditions were detected from ten patients and five therapists: fears, physical limitations, test instruction, testing conditions, and therapists’ expectations. **Conclusions:** Patients with coronary heart disease had poor self-perception of exercise intensity and limited understanding of the meaning of the Borg Scale and the 6-MWT. Physiotherapists should place greater emphasis on patients’ perceived exertion to be able to effectively tailor exercise prescription and, therefore, improve attainment of cardiac rehabilitation goals and long-term adherence.

## 1. Introduction

Cardiac rehabilitation (CR) is the mainstay of secondary prevention in patients with coronary heart disease (CHD). The evaluation of exercise capacity is part of the initial clinical assessment at the start of a CR program. The 6-minute walk test (6-MWT), as a submaximal test, is particularly appropriate to measure exercise capacity in patients with reduced general condition after an acute cardiac event or a medical intervention [1]. It is a widely used, well-evaluated, and easily applicable tool in the elderly [2]. Unlike maximal exercise stress tests on a treadmill or an ergometer, the 6-MWT reflects submaximal functional exercise capacity that is more representative of routine activities, particularly in older adults [3]. It is sensitive to improvements in functional capacity after medical interventions and rehabilitation [4] and has demonstrated significant prognostic value in terms of morbidity and mortality [5]. At the same time, the Borg Scale (BS) is used to assess the subjectively rated perceived exertion (RPE). In recent times, the initially described scale from 6 to 20 has been modified and is now often used with a range between 0 and 10 [6,7]. However, this method lacks proper validity and reliability to determine endurance exercise intensity in CHD patients [8]. The review of Chen et al. showed that it was primarily tested in younger subjects (mean age 32.7 years) [9], which does not correspond to a typically elderly population of CHD patients. Out of 64 studies, only 4 were conducted with healthy subjects in the age range of 61–80 years. The correlation between BS and heart rate was moderate across all age groups (r = 0.62), decreased with a lower fitness level (r = 0.52), and varied for different test forms and types. In an earlier study, the correlation in elderly subjects (50–68 years) was r = 0.6 [10]. Moreover, there is some discrepancy between various studies [11]. On the other hand, the implementation of a learning protocol has been demonstrated to enhance the validity of the BS [12].

In our experience, we often notice a discrepancy between the intensity of RPE of patients and the appraisal of physiotherapists in the sense that patients exert themselves lower than expected. In a population with mobility limitations (mean age 77 years), RPE and gait characteristics (e.g., energy cost of walking) did not correlate with each other (r = 0.01; CI: −0.30 to 0.32) [13]. Furthermore, the rate of agreement between self-report and the performance of ADL measures was associated with a decline from the pre-hospital level of ADL functioning that occurred during hospitalization [14]. Therefore, additional parameters are warranted to better understand the patient’s view.

Many patients feel uncertain about their exercise capacity at the start of a CR program and show difficulties in rating their perceived exertion [15]. This may be due to a reduction of the general state of health or more specific symptoms, such as muscular fatigue, shortness of breath, and chest discomfort [16]. Shea et al. also showed that different recommendations for exercise training, based on target heart rate or RPE, did not successfully reduce fear [17].

Adherence to a CR program, i.e., active participation in exercise sessions and accurate self-assessment of RPE, is crucial for achieving optimal short- and long-term results [18]. Thereby, optimal exercise prescription and guidance of the patients during the initial phase of recovery is important [19]. To date, no data are available regarding the factors influencing the use of the RPE instrument during a 6-MWT, in this context, in particular respecting the patient view. Thus, this study aims to explore low self-rating of exercise intensity during the 6-MWT at the start of an inpatient CR program, with a particular focus on self-assessment.

## 2. Materials and Methods

An explorative, qualitative research approach was chosen to explore influencing factors that may explain low self-rating with the grounded theory method [20]. The present study was conducted from June 2021 to June 2022 at the Clinic Barmelweid, a stationary rehabilitation center in Switzerland. It was approved by the Ethics Committee of Northwestern Switzerland (Project ID 2021-01036) and registered as NCT04898296 (https://clinicaltrials.gov/). The study complies with the Declaration of Helsinki. All participants provided informed written consent.

### 2.1. Inclusion and Exclusion Criteria of the Patients

The estimated sample size of 5–10 patients was chosen to maximize the range of influencing variables that could be identified and used to explain the phenomenon in the interviews. Recruitment was carried out purposively from the institution’s admission list. No incentives were offered or given. Patients were eligible if German was the native language, the age range was between 65 and 85 years, they were able to perform a 6-MWT, and BMI was <30 kg/m^2^ (to avoid obesity as an influencing factor when walking) [21]. The 6-MWT had to be performed for the first time. Patients were included in the study if their RPE was <7 on a scale from 0 to 10. Exclusion criteria were as follows: patients with insufficient linguistic comprehension to participate in an interview or unable to complete a questionnaire, and patients unable to perform a 6-MWT, for example, due to recent surgery on major joints of the lower extremities, unstable heart disease, or predominant neurological symptoms.

The instruction and implementation was carried out according to standardized specifications for each patient individually [22]. The patient was informed that their ability to walk will be tested by means of a walking test. For this purpose, the patient was given an information sheet describing the exact procedure and explicitly mentioning that the patient should try to walk as far as possible. Other test conditions, such as footwear, food intake, and medication, were also discussed. After the test, the patient was given instructions on how to use the BS.

### 2.2. Inclusion and Exclusion Criteria of the Therapists

A focus group of physical therapists can serve as a tool to provide diverse perspectives that challenge the investigators’ potential preconceptions.

These pre-existing beliefs could shape how patients’ experiences and the phenomenon under study are interpreted.

The therapists were purposively selected from a team of twenty employees in the department who work exclusively with patients with CHD. Eligibility criteria were at least 5 years of professional experience and/or further training as a specialized rehabilitation therapist and/or a management function. By assembling a focus group of physical therapists with varied backgrounds, levels of experience, and perspectives, the investigators gained access to a broader spectrum of insights. The group discussion facilitated the identification and examination of alternative viewpoints and interpretations, thus serving as a check against the investigators’ personal estimations. It also encouraged critical reflection on the assumptions embedded in the research process. Physical therapists should also have already carried out regular gait tests in order to be able to reconstruct examples with patients from everyday life. Preference was given to people with professional and organizational leadership who demonstrated an increased awareness of quality aspects of test implementation.

### 2.3. Data Collection

The semi-structured interview of the physiotherapists was conducted first. The data were recorded, in addition to the patients’ data, in order to record other factors that patients may not name.

The aim was to explore factors influencing patients’ perception of exertion during the test, particularly for older patients, and why their self-assessments might differ from therapists’ expectations. Open-ended questions addressed potential reasons for underestimation, the role of therapists’ expectations, the influence of the test procedure, and the implications of these discrepancies for practice or future research.

After successful completion of the 6-MWT, all patients completed a specific questionnaire and underwent a semi-structured interview.

The questionnaire aimed to identify factors influencing their experience and perception during the 6-MWT. It included questions about prior experience with the 6-MWT, their understanding of instructions, perceived exertion using the 0–10 scale, and their ability to provide honest self-assessments. Further questions explored their perception of exertion, willingness to push to their limits, feelings of safety during the test, and factors that might restrict them from reaching their limits. Additional topics included comparisons of physical effort before and after their heart condition, recent experiences of intense exertion, and suggestions for improving the test procedure.

The interview began with an introduction of the interviewer, followed by a reminder that all responses will remain anonymous and be audio-recorded for transcription. The aim was to explore factors influencing patients’ perception of exertion during the 6-MWT. Open-ended questions addressed experiences during the test, the impact of daily physical strain, perceptions of exertion, differentiation between levels of intensity (e.g., light vs. very strenuous), and factors that might influence these perceptions. Patients were also asked to reflect on changes in their exertion levels over time and how they evaluate their effort during physical activity.

After each interview, the influencing factors were coded. A constant comparative process of analysis was used to determine common themes.

### 2.4. Description of the Study Process

Therapists were invited to participate in a separate focus group session (45–60 min). Patients participated individually in an interview (30–45 min).

From the patient and the therapist data, a descriptive evaluation was performed. Data collection was guided by theoretical sampling [20]. Since the perspective of the physiotherapists was only included in the evaluation as a supplement, the recordings from the focus group were only used for the open coding step. Interviews were continued with up to ten subjects to achieve a high degree of saturation. The focus was set on the point that no new codes were generated.

The transcription was made verbatim according to the simple rules, preserving linguistic nuances, e.g., pauses [23]. Conversations in Swiss German were translated into High German. The transcripts were reviewed multiple times, cross-checking them with the original recordings and ensuring that no important information was omitted or misinterpreted. Transcripts of the audio recordings (Olympus WS-853) of the focus group and the patient interviews were analyzed using MAXQDATA (version 2020, VERBI Software, Berlin, Germany). The quotations for the article were translated into English by the first author in a two-language table and proofread by the second author [22].

### 2.5. Coding Process

A 3-step analysis process of the ‘grounded theory method’ was run through. The open coding of the interview texts was performed under the guiding question: ‘What explains low self-ratings of exercise intensity during the 6-MWT?’ All interviews were examined line by line, the data were analyzed in small parts, and thus concepts were created from statements (supported by memos) of the interviews of the patients or the focus group. A coding guide was created (cf. Appendix B). Categories were reconstructed in horizontal coding in terms of their interrelationships with the other categories [24]. In this process, significant concepts and categories were correlated to the other categories within the framework of a coding paradigm. In this way, significant categories could be considered and analyzed as individual delimited phenomena in a causal network of different aspects—characteristics, causes, intervening conditions for strategies with the phenomena, and consequences. The axial coding of the categories represented a systematic approach for an explanation of the main phenomenon under investigation, namely, the lower subjective assessment in the 6-MWT in the special situation of inpatient cardiovascular rehabilitation following an acute event.

Selective coding was the final step in the evaluation process of the grounded theory method to provide a comprehensive answer to the research question raised at the outset.

## 3. Results

### 3.1. Results of the Physiotherapists

Five physiotherapists (28–33 years; 1 male) participated in the focus interview (47 min). They had between three and eleven years of professional experience. The open coding of the statements within the focus group resulted in a separate main category on the therapists’ expectations (cf. Appendix B).

The physical therapists’ expectations of the patients’ assessments after the 6-MWT were associated with a high score on the BS (7–10), as described by one therapist:

“But it just doesn’t match what we expect through the test. So, I already find that somebody can recognize that the patient is actually not exerting to a high degree. And often, when I get a three or a four, I think: Yes, you look like that (...). But it doesn’t correspond to what we would like.”

(Female, 31 years; 6 years of experience.)

### 3.2. Results of the Patients

Six women and four men were consequently included, as they scored low (0–6) on the BS during the 6-MWT, with a balanced proportion to include both genders. The duration of the interviews was between 28 and 49 min. The patient characteristics are summarized in Table 1 and Table 2. The results of the questionnaire can be found in Appendix A.

### 3.3. Results of Open Coding of Both Groups

From the interview statements, 357 codes were formed, which were summarized in 38 categories (Appendix B). Of particular note was the acceptance of reduced activity levels by patients and the expectations of physiotherapists with regard to the evaluation of patients’ perceptions of the intensity of the exertion.

### 3.4. Summary of the Conceptual Categories

The overarching themes that emerged from the interviews and the focus group included physical limitations, fears, instructions, test conditions, and self-image of patients with CHD at retirement age. These themes were created from statements.

### 3.5. Physical Limitations

This category was responsible for a low covered distance, which was the reason why patients also tended to give their BS a low rating. They frequently mentioned physical symptoms caused by the walking test, such as pain, shortness of breath, leg fatigue, lack of sleep, and/or dizziness.

### 3.6. Fears

In the main category of fears, patients’ statements were included that referred to potential negative events, dangers, or negative feelings, and the associated restraint of the patients. The 6-MWT, or the request to walk as far as possible, generated fears among patients regarding potential negative consequences:

“Well, I wouldn’t have dared to set the tempo in such a way I would have been capable for fear of burning muscles.”

(No. 1; 80 years old, male, after bypass operation (BO).)

### 3.7. Instruction

In this category, statements from the patients on how they followed the instructions for the 6-MWT and the BS were collected. Not only was the understanding of instructions a prerequisite for perceiving exertion intensity, but also how the perception translated into an adequate value:

“You have to get mentally into that scale first.”

(No. 2, 66 years old, male, after percutaneous transluminal coronary angioplasty.)

After the instruction of the walking test, the patients had to determine how fast they walked and rate their speed. One patient stated that he chose a tactic of not giving the maximum at the beginning (No. 8, 75 years old, male, after BO), and that the rhythm of walking was important for him. Another patient described that he alternated the speed, but it was important for him to feel comfortable.

### 3.8. Test Conditions

From the patients’ point of view, the 6-MWT should be well integrated into the daily routine and coordinated with other physical therapies in order to avoid stressful situations.

The importance of the test was not so relevant for patients, but they recognized that it was more important for the therapists:

“But I know that the test is not for me, it’s for you or your work and it can be very helpful. Right? But for me personally, it doesn’t matter, does it?”

(No. 8.)

Some patients also commented on the low relevance of the BS for their everyday life:

“(...) when I do this exercise [the 6-MWT], I know now that I have to make an effort to get there and to get an effect then. But in everyday life, I don’t think so.”

(No. 1.)

Furthermore, the content-related test conditions were influenced by the importance of patients according to the perceived intensity in itself. In their statements, it turned out that it was not important for their everyday life:

“Yes, but I don’t really ask myself that. The work, does it bother me now or not. I just have to get it done.”

(No. 1.)

### 3.9. Self-Image at Retirement Age in Coronary Heart Disease

Patients frequently linked their self-image, their age, and heart disease regarding their perception of stress:

“So, I think what changes the most [with age] is the serenity, just not getting excited so quickly, letting it be what it is. I don’t think it was like that in the younger years, but now it’s serenity, enjoying. Yeah, I think that’s changing—to be satisfied. You’ve had life for the most part; now there’s the beauty to come (...)”

(No. 7, 67 years old, male, after BO.)

In terms of self-perception of their own limits of performance and activity levels, the patients stated that they only wanted to be moderately physically active:

“So, with Nordic walking, initially I could always keep up with all the participants. And then, lately, I noticed that I was struggling uphill. And then I started to fall behind. Maybe I could have tried harder, but it was also something indeterminate, wasn’t it? And then I didn’t totally push the envelope either, did I?”

(No. 1.)

The patients also stated that they felt a slow physical decline with advancing age. However, they perceived the changes in their musculature and circulation as positive, knowing their limits, and adapting their activity to these limits. They described performing activities frequently within the well-being range:

“Normally, the effort should be at most moderately difficult. You should also be able to enjoy it. That’s actually the goal. Good on foot, not too heavy, enjoy and still do something for your health.”

(No. 6, 75 years old, male, after BO.)

Three patients described an acceptance of decreasing activity in old age and—according to their event of coronary heart disease—a lack of ability to assess their exercise capacity. They compared their current with their previous state and indicated uncertainty in adequately assessing themselves, e.g., about previous training.

The patients described a low score on the BS related to their age-related self-image. One patient explained the statement as follows:

“So, when I’m really at the limit, then I say six or five, (...) it’s [the] maximum, (...) a limit has been reached, so I really can’t go any further. But (...), that’s not absolute, so in the past, I might have been able to do a lot more and now it’s just within the framework, the limit, right?”

(No. 2.)

### 3.10. Axial and Selective Coding

The axial coding paradigms showed that the category of self-image at retirement age in CHD had a multifaceted relationship with patients’ perceptions of their limitations and their associated fears.

**Figure 1:**
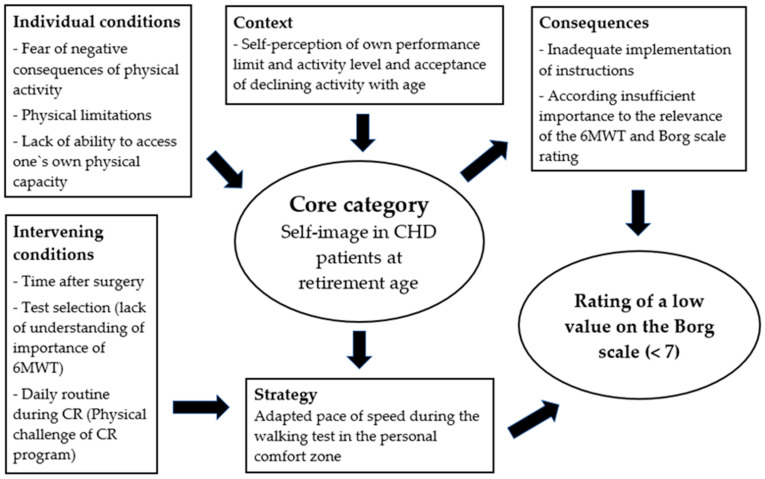
Coding paradigm core category “self-image in coronary heart disease (CHD) at retirement age”.

In order to provide an explanation for a low BS rating after a 6-MWT, the interdependence of the core category “self-image at retirement age” of CHD patients and influencing factors was analyzed (Figure 1). On the one hand, fear of negative consequences of physical activity, physical limitations, and lack of ability to access one’s own physical capacity influenced the self-image of CHD patients. This led to an adapted pace of walking speed, where the patients remained in their personal comfort zone. This, in turn, was significantly influenced by the time after surgery, the understanding of the test requirement, and the unfamiliar daily routine of a CR program. All these aspects may hinder patients to adequately implement instructions or give enough weight to the 6-MWT and BS rating, finally leading to the reporting of a low BS.

## 4. Discussion

The present study investigated the subjective perception of exertion in the 6-MWT in patients of retirement age with CHD. The background of the study was that physiotherapists instructing the test noticed that the patients usually rated themselves lower than expected. The main result of the study was that the self-image of these patients was the key category able to explain the low rating on the BS.

The importance of self-image by aging is crucial in understanding functional outcomes in older adults, as demonstrated by their strong influence on health trajectories [27]. The perception that aging is associated with physical losses leads to lower use of strategies promoting a healthy lifestyle [28], as shown in this study with the lower pace of the speed during the walking test. Additionally, images of old age shape our behavior toward aging and have an impact on our self-image [29]. Negative self-perceptions of aging (e.g., acceptance of declining activity with age) impair the use of adaptive strategies like selection, optimization, and compensation, leading to poorer functional outcomes, while positive strategies promote better health and life satisfaction after serious health events [28]. Particularly in younger elderly people, acceptance of aging was negatively related to quality of life [30]. The self-perception of aging also affects self-efficacy, which in turn probably motivates people to use effective coping patterns for maintaining physical functioning [31].

The perception of exercise intensity at retirement age should be subject of more research in order to optimize recommendations, explanations, and types of implementation for exercise intensity, and finally to improve adherence with the WHO recommendations for health-promoting activities [32] for patients in CR [33].

Our patients’ statements about their fears in the face of physical strain are consistent with those in the study by McCormack et al. [34]. In contrast, Muotri et al. noted that patients with panic disorder were negatively affected by breathlessness during an ergospirometric challenge and rated higher than a control group [35]. Fear is projected to the future and often described in psycho-oncology [36], but it is also prevalent in cardiac patients with health-related issues and associated with avoidance in a test situation, such as the 6-MWT. Patients feared that engaging in physical activity might induce a future cardiac event or damage their heart [36].

In order to minimize anxiety and fear after an intervention (e.g., surgery or myocardial infarction), it is important that patients are accompanied closely by a cooperating team of therapists, i.e., that the same person accompanies the same patient. This can impose a challenge in day-to-day operations due to limitations in staff. The possibility of a rehearsal of a walking test or a repetition could lead to more certainty but is time-consuming. A second implication is to possibly select a different test for the initial assessment, e.g., climbing stairs, as suggested by one patient. However, this would have the disadvantage that some patients would have to take breaks. An alternative would be the widely used incremental shuttle walking test [37], in which the speed requirements increase slowly. A review of this test in patients with heart failure confirmed that it could generate higher intensities [38].

Integrating the BS in a submaximal endurance test (e.g., the self-paced 1 km treadmill test) may allow for real-time adjustments of walking speed based on patients’ perceived exertion [39]. This dynamic approach could help identify discrepancies in perceived exertion more accurately and provide tailored feedback for both exercise prescription and patient education.

While the 6-MWT is not inherently designed as an educational or motivational instrument to directly influence patients’ lifestyle habits, it does offer an opportunity for patient self-assessment. This should be emphasized more strongly to the patient in order to avoid the impression that the 6-MWT is only important for the therapist. By comparing their performance to established normative values [25], patients can gain insight into their functional status and thereby understand their physical capabilities relative to their peers.

The repeated measurement of the 6-MWT (at the start and at the end of the CR program) may also allow patients to track the improvement or the decline in their performance. This visual and measurable feedback may have an educational impact on the effectiveness of the rehabilitation program and/or subsequent lifestyle changes, fostering a sense of achievement and reinforcing their commitment to physical activity.

As a sensitive tool to detect functional impairments that may not be apparent in routine clinical examinations [22], the 6-MWT may help healthcare professionals to educate patients on specific challenges they face and to tailor interventions according to the amount of deficit and underlying clinical limitations, taking into account the patients’ beliefs about the reasons for their physical limitations.

How to familiarize older people with the BS is well described in the literature and could be applied in CR. In active therapies, physiotherapists would have the opportunity to reduce and overcome anxiety [40] and adapt activities to physical limitations. Patients should be educated as to why higher intensities are beneficial to their health [32]. Therefore, strategies are needed to communicate to older patients to perform activities at a certain intensity and move out of their comfort zone, manage anxiety and expectations in order to achieve effective changes in their health status.

Our patients in the age group between 65 and 85 years could not or did not want to exert themselves in a high range of the BS. They accepted their declining activity with advancing age. Van der Wahl et al. [41] similarly found that approximately 50% of patients with heart failure did not adhere with the recommendation for a minimum level of physical activity, resulting in a significant lack of activity, which led, similar to the patients in the present study, to an acceptance of decreased activity. Of note, a lowered and decreasing level of physical activity in patients over 65 years of age is associated with a higher rate of adverse cardiovascular events [42].

Safety during physical activities and fear of falling are independently seen as potential barriers to desired physical activity behaviors in older adults [43]. Accordingly, patients in the present study reported special caution in activities, such as choosing only paved paths when bicycling or using poles when walking, and taking more time in their activities, which reduced the intensity. A pilot study that sought to improve intensity through the use of pedometers in a group of patients with an average age of 72 found only an increase in exercise duration but not in the intensity of a moderate to high-intensity range [44]. They accepted their performance limitations and liked to be active in their comfort zone—for safety reasons and to reduce anxiety [40].

Patients’ interpretations of the BS were affected by their understanding of the scale’s verbal descriptors, which could lead to a misalignment between their RPE and the scale’s numerical values. Although the BS is used often in older adults, a cross-cultural adaptation process was not performed [45]. Betablockers could not explain the low expression of exertion, as patients on cardioselective betablocker therapy produced similar exercise intensities compared with cardiac patients who were not receiving betablocker treatment [46]. Validation studies are lacking in this area, but there is cross-cultural data in the literature on how to familiarize older adults with the BS [47].

Also noticeable was a conscious acceptance of declining activity in old age. This contributed to the fact that the patients did not go to their limits in the test situation and, therefore, did not fulfil the expectations of the physiotherapists. For patients, it was important to feel comfortable during physical activities, which went hand-in-hand with a low intensity of exertion. They attached little importance to the BS and the 6-MWT. To counteract these beliefs, increased education, as well as an active treatment program, as offered during CR, are needed to help patients to better manage self-imposed limitations and anxiety. As discussed above, there are a number of factors that may play a role in maintaining regular physical activity and choosing an appropriate intensity. In this context, at the end of the 6-MWT, the physiotherapist has a unique opportunity to reflect with the patient on the lower subjective rating compared with their own impression, to explore possible reasons, such as anxiety or physical limitations, and to target them individually. Applying such reflections, not only after a 6-MWT but also during or after further exercise sessions, could help to improve adherence to the recommended exercise prescriptions and thereby long-term exercise performance.

### Limitations

It is worth noting that the study’s small sample size and its focus on a specific demographic (retired patients with CHD in an inpatient CR program) could limit the generalizability of the findings. The study did not consider differences in age, gender, or type of CHD between patients (mean age was 74.5 years, with 6 males and 4 females) or physiotherapists (age 28–33 years, with 1 male and 4 females) with regard to years of experience or working style. However, the qualitative nature of the study provided valuable insights into the multifaceted aspects of RPE among this specific population. The small sample size may limit the ability to identify consistent or robust patterns, especially in qualitative studies seeking theoretical saturation.

However, no contrasting cases could be collected in the theoretical sampling that could falsify the theory, e.g., patients who rated themselves in the upper range on the BS. The validity would also have been increased if subjects from other inpatient or even outpatient rehabilitation centers would have been included in the study. The validity of the present theory is thus applicable to a narrow group of patients, limited by age, type of disease, and setting of an inpatient CR program. A homogeneous group was sought in order to carry out a typification. Therefore, it cannot be transferred to other groups, e.g., those having a different sociocultural background, insufficient linguistic comprehension, or obesity.

## 5. Conclusions

In the present study, we showed that self-image was the key category able to explain a low rating with the BS during a 6-MWT.

Therefore, physiotherapists should pay more attention to the patients’ RPE during rehabilitation to better guide patients, e.g., during endurance training at a targeted intensity. They have to improve the test instruction and conduct explanatory sessions prior to the 6-MWT, where patients can practice the application of the BS in simulated activities.

The patients’ self-perception of exercise intensity and their understanding of the importance of the BS, as well as further research with larger and more diverse samples of patients, could corroborate and expand these findings, enhancing our understanding of the complex dynamics involved in patients’ RPE during a 6-MWT [48].

## Figures and Tables

**Table 1:** Patient characteristics.

Characteristics	Measurement
N (male)	10 (6)
Mean age, years (range)	74.5/(67–82)
Mean BMI, kg/m^2^ (range)	23/(18–29)
Time to the acute event, days in preceding hospital, mean (range)	11.5 (8–20)
Previous myocardial infarction	4
NSTEMI	1
STEMI	3
Condition after reanimation	1
Degree of coronary heart disease	
2-vessel disease, n	6
3-vessel disease, n	4
Intervention	
PTCA with stent	4
Coronary bypass graft surgery	5
Comorbidities	
Heart failure	1
Diabetes mellitus	4
Arterial hypertension	5
Atrial fibrillation	4
Patients with betablockers	10
Civil status	
Living in partnership	5
Living alone	5

BMI: body mass index; PTCA: percutaneous transluminal coronary angioplasty; STEMI: ST-elevation myocardial infarction; NSTEMI: non-ST-elevation myocardial infarction.

**Table 2:** Results from the 6-MWT.

Variable	Measurement
Mean distance, m (range)	401 (168–553)
Percentage of predicted value, m (range)	80 (37–101)
Difference between heart rate before/after test, median (minimum/maximum)	15/(1–35)
Percentage of predicted value, heart rate (range)	65 (41–72)
Value of perceived exertion (score 0–10)	
**Value**	**Number**
1	1
2	1
3	1
4	3
5	4

See references for equations of Enright [25] and Inbar [26].

## Data Availability

The data presented in this study are available upon request from the corresponding author due to storage in a special software.

## Appendix A. Results of the Questionnaire of the Patients

Instruction and Understanding

Concerning the understanding of the instruction on the 6-MWT, six patients stated that they had understood the instruction to exert maximum effort, two others ticked that they had partially understood this, while two patients stated that they had not understood this instruction at all. Three patients ticked that they had not dared to exert maximum effort (question 2). The patients confirmed that the BS had been explained to them (question 3a) and that this explanation had been understandable to them (question 3b), but two of the patients thought it would have been helpful to explain it to them in more detail (question 3c). All but one patient thought that the time to assess themselves had been sufficient (question 3d), and two patients thought it would have been helpful to have the BS explained to them beforehand (question 3e).

Honesty

- Four patients reported that they were unable to reliably assess themselves in terms of indicating exercise intensity, e.g., due to their “heart surgery” and in terms of “relation” (i.e., to their previous activity level; question 4).

Perception

- One patient stated that effort was difficult to assess after “surgery”. Five patients perceived exertion concerning breathing, three related it to heartbeat, and others associated exertion with activities when “walking” or “riding a seated bicycle”. Another patient perceived fatigue (question 5—free text).

Self-Assessment of Physical Activity

- When asked whether they had pushed themselves to the limit before the cardiac event, the patients rated themselves on a scale of 0 to 10 with values between 2 and 10 ($\bar{x}$ = 6.2; 0 = rather no; 10 = rather yes). After the acute event of heart disease, this assessment was significantly lower in five subjects than before the event ($\bar{x}$ = 5.2; minimum: 0; maximum: 10; question 6).

Security

- The feeling of being safe during the 6-MWT was reported differently by the patients on a scale of 0 to 10, and the mean value was $\bar{x}$ = 7.5 (minimum: 0; maximum: 10; question 7). In three patients, the value was less than five.

Physical Performance Limit

- The patients were able to indicate in free text what would generally prevent them from reaching their performance limits. For example, they named physical symptoms, such as “tiredness”, “pain”, “shortness of breath”, “dizziness”, or “the ability to walk”, as well as “their current state of health” (question 8).
- As a restriction to reaching their own performance limit in the current situation, the patients listed, for example, keywords related to “heart rate” as well as “pain”, “operations”, “fear”, “restlessness”, and “an uncertainty due to illness” (question 9).

Self-Assessment as a Sporty Person

- The answer to the question of to what extent the patients considered themselves to be a person who is active in sports on a scale of 0 to 10 was rated very differently, with a mean value of $\bar{x}$ = 5.0 (minimum: 0; maximum: 8; question 10).

Maximum Effort and Limits in Physical Activity

- As strong stressors before and after the acute phase of the heart disease, the patients named sports activities such as cycling or running, but also everyday activities such as climbing stairs and walking (question 11; only one respondent’s answer was inconclusive, as she entered a value instead of an activity).
- When asked how long it had been since they had exerted themselves to the maximum during an activity, four subjects stated that it had been longer than a year ago, and three of the patients wrote that it had been several years ago. Three patients answered that it had been a few weeks ago (question 12).
- Patients cited the following as reasons for not being able to continue to exert themselves in the test: “previous traffic accident”, “no need”, physical symptoms, such as “breathing” and “weakness”, but also “insecurity” and “age” (question 13).

## Appendix B

**Table A1:** Coding guide.

Main Category	Definition	1. Subcategory	Definition	2. Subcategory	Definition
Self-image at retirement age in CHD	The idea that patients with CHD have of themselves is based on self-image, which controls thinking, feeling, and behavior	Self-perception of own performance limit and activity level	Assessment of the current activity level is related to the experience of the previous activity level and performance (current due to illness or months and/or years ago)	In the well-being area	Offers security and feels comfortable, e.g., breaks set by yourself
				# An acceptance (or lack thereof) of declining activity in old age	The limits of physical activity are (not) accepted
				Influenced by a lack of ability to assess one’s capacity	The patients express that they have difficulty assessing their capacity
				Evaluation of self-perception not comprehensible by therapists	Patients give a different assessment of self-perception—different from the assessment of others
				Indication of a low value on the Borg Scale, below seven	Patients state that they cannot/do not want to burden themselves any further
		Due to the influence of the outside	Patients are influenced by their social environment or do not like to show up in public when they walk slower or need aid		
Physical limitations	Physical limitations that the patient perceives	Due to the effects of CHD, including the consequences of surgery	Signs and symptoms of coronary artery disease and/or sequelae of surgery		
		Due to an age-related, decreasing fitness level	Reduction of muscle strength, endurance, and balance		
		By secondary diagnoses	Diagnoses other than coronary artery disease that relate to exercise capacity		
		Through the physical sensation of stress	Loads that arise physiologically		
		* Through hygiene regulations	Patients must wear face masks due to COVID-19 regulation		
Fears	Any possible negative events, dangers, or negative feelings that could result from the test and lead to the patient’s restraint	Insecurity	Patients experience feelings of insecurity because they are not able to adequately process or interpret the current (disease) situation		
		Anxiety	Patients perceive stress as threatening		
Instruction	Give instructions on the walking test and the effort scale	Receptivity	Ability to cognitively absorb information		
		Implement instructions	The ability of the patients to implement the instructions adequately	# Influenced by unclear or misunderstood physician guidelines	Load specifications or restrictions are not communicated or included
				Influences the classification of the speed during the walking test	Assumption of how the instruction is translated into a self-selected speed
		* Interaction: therapist–patient	Statements about the interaction between the therapist and patient during the test		
		Lack of instruction in the test itself	The patient is not sufficiently informed about the test		
		Timing of the instruction for the load intensity	Instruction of the Borg Scale before the test		
Test conditions	All organizational, content-related, spatial, and personal aspects necessary for the implementation of the 6-MWT and application of the Borg Scale				
		(A) Organizational		Query timing	When is the load intensity inquired?
				Daily schedule	Time of the test and general load during the day
				* Adherence to the dates	“Slow” and indecisive patients go beyond the scheduled test appointment time
				Time after surgery	An earlier or later date for scheduling the walking test after surgery
		(B) Content		* Learning effect	Improvement through repetition
				Test selection	The test is not suitable for the subjects
				Meaning of the test	The 6-MWT has relevance for patients
				The low importance of the Borg Scale	Use of the Borg Scale in everyday life is low
				Influenced by the importance of the load intensity	The patients do not rate the intensity as important for their everyday life
		(C) Spatial		Surroundings	Interference from facilities or persons presents other than the therapist in charge
		(D) Under medication		Test under medication	Medications that alter the capacity
* Expectations of the therapists	Evaluation of the patients’ assessments of the intensity of the load				

(Those marked with * were mentioned only by the focus group, and # only by the patients).
